# Dyskerin Mutations Present in Dyskeratosis Congenita Patients Increase Oxidative Stress and DNA Damage Signalling in *Dictyostelium Discoideum*

**DOI:** 10.3390/cells8111406

**Published:** 2019-11-08

**Authors:** Javier Rodriguez-Centeno, Rosario Perona, Leandro Sastre

**Affiliations:** Instituto de Investigaciones Biomedicas, CSIC/UAM and Centro de Investigación Biomédica en Red de Enfermedades Raras, CIBERER, 28029 Madrid, Spain; biojaroce@gmail.com (J.R.-C.); rperona@iib.uam.es (R.P.)

**Keywords:** dictyostelium, dyskerin, dyskeratosis congenita, telomere, telomere biology disorder, DNA damage, oxidative stress, pseudouridylation

## Abstract

Dyskerin is a protein involved in the formation of small nucleolar and small Cajal body ribonucleoproteins. These complexes participate in RNA pseudouridylation and are also components of the telomerase complex required for telomere elongation. Dyskerin mutations cause a rare disease, X-linked dyskeratosis congenita, with no curative treatment. The social amoeba *Dictyostelium discoideum* contains a gene coding for a dyskerin homologous protein. In this article *D. discoideum* mutant strains that have mutations corresponding to mutations found in dyskeratosis congenita patients are described. The phenotype of the mutant strains has been studied and no alterations were observed in pseudouridylation activity and telomere structure. Mutant strains showed increased proliferation on liquid culture but reduced growth feeding on bacteria. The results obtained indicated the existence of increased DNA damage response and reactive oxygen species, as also reported in human Dyskeratosis congenita cells and some other disease models. These data, together with the haploid character of *D. discoideum* vegetative cells, that resemble the genomic structure of the human dyskerin gene, located in the X chromosome, support the conclusion that *D. discoideum* can be a good model system for the study of this disease.

## 1. Introduction

Dyskerin is a nuclear protein highly conserved in archaea and eukaryotes. Dyskerin associates with H/ACA small nucleolar RNAs (snoRNAs) and with small Cajal Body RNAs (scaRNAs) to form ribonucleoprotein complexes (snoRNPs and scaRNPs). Dyskerin always is associated in these RNP complexes with other three highly conserved proteins, NOP10, NHP2 and GAR1 [1]. snoRNPs participate in several biological processes. Dyskerin has pseudouridine synthase activity and is responsible for the modification of uridine residues in different RNA molecules, including ribosomal RNAs (rRNAs), small nuclear RNAs (snRNAs) and messenger RNAs (mRNAs) [2]. The specificity of the reaction, including the RNA molecule and the residue modified is determined by the associated snoRNA that acts as a guide [3]. Pseudouridylation is required for the proper structure and function of these RNAs. In addition, H/ACA snoRNPs direct endonucleolytic processing of rRNA [4], enforcing the role played by dyskerin in ribosome biogenesis. The subgroup of H/ACA snoRNAs that localize to Cajal bodies target small nuclear RNAs (snRNAs) of the U1 spliceosome [5]. In addition, snoRNAs can be processed to short regulatory RNAs that modulate alternative splicing [6] or to microRNAs (miRNAs) that regulate gene expression [7].

Dyskerin and their associated proteins also interact with the RNA component of the telomerase complex, named telomerase RNA (TR, encoded by the *TERC* gene), which harbors a H/ACA domain in the 3′ region [8]. In fact, dyskerin was identified as a component of the telomerase active holoenzyme [9]. Determination of the structure of the human telomerase holoenzyme has shown that two dyskerin-NOP10-NHP2-GAR1 complexes associate to a single TERT-TR complex in substrate-bound enzyme [10]. Telomeres are nucleo-protein structures present at the end termini of chromosomes. Their DNA sequence is composed by repetitive sequences (TTAGGG repetitions in humans) and is associated with a protective protein complex such as the shelterin complex present in vertebrates (reviewed by [11]). Telomeres cannot be completely synthesized during DNA replication and become progressively shorter during cell proliferation, which would finally result in genomic instability cell senescence or apoptosis [12]. Therefore, telomeres are extended by specific mechanisms in proliferative cells. The most common reaction of telomere extension depends on the reverse transcriptase activity of the telomerase complex that uses the TR molecule as a template [13].

Sequence analysis identified three conserved functional domains in dyskerin [14]: the dyskerin-like domain (DKLD), implicated in RNA binding [10]; the TruB_N pseudouridine synthase catalytic domain and the pseudouridine synthase and archaeosine transglycosylase (one enzyme required for the synthesis of archaeosine, a 7-deaza guanine derivative present in most archaeal tRNAs) (PUA) RNA binding domain. Most metazoan homologous proteins contain a N-terminal extension [15] and two nuclear localization signals (located at the N- and C-termini) involved in nuclear and nucleolar localization (reviewed by Angrisani et al. [14]). Mutations in the gene coding for human dyskerin (*DKC1*) produce the rare disease X-linked dyskeratosis congenita (X-DC). This is a complex disease that usually produces a classical triad of mucocutaneous features: nail dystrophy, reticular skin and oral leukoplakia, but also many additional symptoms such as bone marrow failure, pulmonary fibrosis, premature aging or increased tumor susceptibility [16,17]. Dyskerin mutations found in patients correspond to hypomorphic or gene silencing mutations since this gene is indispensable for survival in the organisms presently studied [14,18,19]. Most of these mutations are located to the N-terminal and C-terminal domains of the protein that wrap around each other in the three-dimensional structure of the protein [20].

Primary fibroblasts and lymphoblasts from X-DC patients have shorter telomeres than age-matched control cells and lower levels of telomerase RNA (TR) and telomerase activity [21]. The study of patients with autosomal forms of dyskeratosis congenita (DC) has identified mutations in other genes coding for proteins involved in telomere biology, including the dyskerin-associated NOP10 and NHP2 proteins, but also components of the telomerase complex (TERT, TERC) or the shelterin complex (TINF2), among others. These mutations cause short telomeres, compared to age-matched controls (reviewed by [22]). These studies have established a strong association between DC, compromised telomerase function and telomere shortening.

*Dictyostelium discoideum* is a eukaryotic amoeba phylogenetically related to both fungi and animals. This organism is used as a model to study many molecular pathways that are closely related to those of mammalian cells [23,24]. It has also been used for the study of pathologic mechanisms of human diseases [25]. *D. discoideum* telomeres are formed by repetitions of A(G)_1–8_ sequences and their maintenance is dependent on the presence of the *terthp* gene [26]. This gene codes for a protein homologous to telomere reverse transcriptases from other organisms. Moreover, *D. discoideum* nola4 (Nucleolar protein family A, member 4) gene codes for a protein highly similar to dyskerin, as shown in the Results section. These similarities to the human telomere replication system make of *D. discoideum* a suitable model system to study dyskeratosis congenita. In addition, *D. discoideum* grows as haploid cells what increases the resemblance to X-linked disorder in human males, as is the case of X-DC caused by mutation in the *DKC1* gene.

In this article we have reproduced two human dyskerin mutations in the endogenous *D. discoideum* gene. The resultant mutant strains show proliferation defects and increased DNA damage and oxidative stress, phenotypes also found in cells isolated from DC patients, and can be considered a good model for the study of pathological mechanisms and possible therapies for this disease.

## 2. Materials and Methods

### 2.1. Cell culture and Transformation

*D. discoideum* cells were grown axenically in HL5 media under shaking (150 rpm) at 21 °C. Cells were alternatively grown feeding on *Klebsiella aerogenes* over SM-agar plates. Cell proliferation was determined by seeding 3 × 10^5^ cells/mL in HL5 media. At the indicated times of culture, cells were counted in triplicate using a hemocytometer and the number of population doublings was calculated. Cells were transformed by electroporation as previously described [27]. Transformed cells were selected by culture in HL5 media in the presence of blasticidin or G418.

### 2.2. Generation of dkc1 Knockin Mutant Strains.

*HindIII*/*XbaI* flanked-*dkc1* gene was cloned into the pGEMTeasy vector, using the primers DKC1F and DKC1R (Table 1). This plasmid was used as template to achieve the Ile23Thr and Thr33Met mutations, replacing 203T>C or 233C>T and 234T>G by site-direct mutagenesis using the primers Mut1dkc1F, Mut1dkc1R and Mut2dkc1F, Mut2dkc1R (Table 1).

The generation of knockin vectors was accomplished using the pLPBLP vector, Cre-loxP system based, with resistance to blasticidin [28]. The 5′ targeting arm was achieved using the primers arm1F and arm1R (Table 1) that incorporated the *ApaI* and *SalI* target sites to facilitate directional cloning into the pLPBLPvector. It covered the −1411 upstream to the −358 to the +1 of *dkc1* gene. The 3′ arm covered the −358 to the +1 using the primers arm2F and arm2R (Table 1) that incorporated the *PstI* and *HindIII* target sites and the *HindIII*/*XbaI* fragments containing the mutated *dkc1* gene. Target sites into 3′arm were finally *PstI*/*SpeI* (*XbaI* compatible).

AX4 cells were electroporated with 10 μg of *ApaI*/*NotI* (New England Biolabs, Ipswich, MA, USA) digested vector. Colonies resistant to blasticidin were collected. Genomic DNA from colonies was isolated using MasterAmp Buccal Swab DNA Extraction Solution (Epicentre, Madison, WI, USA). Successful homologous recombination was validated by PCR reactions with two pair of primers: (1) DKCK5, Bsr; (2) SeqF, SeqR (Table 1). The first pair was designed on blasticidin resistance cassette and upstream to −1411 to evaluate correct insertion. The potential mutated fragments were amplified and sequenced with the second pair. Blasticidin resistant knockin mutants *dkc1*(I23T) (knockin1) and *dkc1*(T33M) (knockin2) were electroporated with pDEX-NLS-cre [28]. After the 24 h recovery period, the cells were placed in axenic medium containing G418 to select for pDEX-NLS-cre transformants. The cells were then plated on SM agar plus *Klebsiella aerogenes* plates for clonal isolation. The clonal cell lines were subsequently picked onto different plates containing axenic medium and blasticidin. All *Dictyostelium* cells grew on SM plus *Klebsiella aerogenes* plates, but only strains that have retained the blasticidin resistance *cassette* grew rapidly in the presence of blasticidin.

### 2.3. DKC1 and Nucleoli Localization

For DKC1-GFP localization, the *dkc1* gene was cloned into the pDV-cGFP-cTAP vector [29] using the *HindIII* and *XbaI* restriction enzymes. 1 × 10^6^ AX4 cells were transfected by electroporation and G418-resistant cells selected. Transfected cells were fixed with paraformaldehyde 3.7%, incubated with DAPI 1:500, mounted on Prolong and visualized with a confocal microscope LeicaTCS SP5 (Leica Microsystems, Wetzlar, Germany).

For nucleoli staining, 1 × 10^6^ methanol (−20 °C)-fixed AX4 cells were incubated with SYTO^®^ RNASelect Green Fluorescent Cell Stain 500 nM (Thermo Fisher Scientific, Waltham, MA, USA) according to the manufacturer´s instructions for 20 min at RT. After being washed twice in PBS, were incubated with DAPI 1:500, mounted and visualized as indicated above.

### 2.4. Pseudouridylation Assay

Total RNA was isolated from 1 × 10^7^ AX4, *dkc1*(I23T) (knockin1) and *dkc1*(T33M) (knockin2) cells using the TRI-reagent (Sigma-Aldrich Inc. Missouri, USA) according to the manufacturer´s instructions. Pseudouridylation assay was performed as described previously [30] with minor modifications. The dried RNA (8 µg) was treated with 30 µL of buffer BEU pH 9 (urea 7 M, EDTA 4 mM, Bicine 50 mM, pH 8.5) or BEU Buffer-CMCT 0.17 M (*N*-Cyclohexyl-*N*′-(2-morpholinoethyl)carbodiimide metho-*p*-toluenesulfonate) (Sigma-Aldrich, St. Louis, MI, USA) at 37 °C for 20 min. Reaction was stopped with 100 µL of buffer A (sodium acetate 0.3 M pH 5.6; EDTA 0.1 mM) and 700 µL EtOH. After being chilled for 5 min in dry ice, the pellet was recovered (14,000 rpm, 10 min, 4 °C), washed with 70% EtOH-water, dissolved in 100 µL of buffer A, and precipitated with 300 µL of EtOH. After being washed, the pellet was dissolved in 40 µL of Na_2_CO_3_ 50 mM pH 10.4, EDTA 2 mM and incubated at 37 °C for 3 h. The RNA was precipitated by addition of 100 µL of buffer A and 700 µL of EtOH on dry ice for 5 min. Pellet was recovered (14,000 rpm, 10 min, 4 °C) and washed with 70% EtOH-water, dried and dissolved in 40 µL of water RNase-free. cDNAs were generated from 500 ng of RNA using random primers and M-MLV reverse transcriptase (Promega, Co., Madison, WI, USA). Quantitative PCR was performed as previously described [31] using the Power SYBR PCR mix (Applied Biosystems, Foster City, CA, USA) and the CFX96 Real-Time System (BioRad, Hercules, CA, USA) with the PCR conditions as previously described [32]. The oligonucleotides for the amplification of *sno18* and *26srRNA* are shown in Table 1. Relative gene expression quantification was calculated according to the comparative threshold cycle method (2^−ΔΔCt^) and CMCT-untreated samples were used to normalize pseudouridylation levels.

### 2.5. Quantitative PCR

Total RNA was obtained from 2 × 10^7^ *D. discoideum* cells. cDNAs were generated from 2 μg of RNA and qPCR was performed as described above. The primers used to evaluate expression levels of *dkc1*, *gar1*, *nhp2* and *nop10* are described in Table 1. Large mitochondrial ribosomal RNA (*lmtrRNA*) was amplified as loading control. Relative gene expression quantification was calculated according to the comparative threshold cycle method (2^−ΔΔCt^) and normalized to the amplification signal obtained for AX4 cells for each mRNA.

### 2.6. Southern Blot

DNA was isolated from 3 × 10^7^ cells of the AX4 and mutant strains as previously described [33]. One microgram of each DNA was either digested or not with 10 units of the *NheI* restriction enzyme (New England Biolabs, Ipswich, MA, USA) at 37 °C for 2 h. Digested DNAs were electrophoresed in 1.2% agarose gels and transferred to Z-Probe membranes (BioRad Laboratories, Hercules, CA, USA). Blots were hybridized to a T(C)n oligonucleotide complementary to the telomeric region [26]. The blot was washed and re-hybridized to a control oligonucleotide complementary to the *26SrRNA* gene, as previously described [26].

### 2.7. DNA Damage

5 × 10^6^ AX4 or dyskerin-mutant cells were treated with 5 µg/mL of Bleomycin for 3 h. γH_2_AX levels were determined by Western blot using Phospho-Histone H2A.X-P-Ser139 antibody 1:1000 (Cell Signaling Technology, Danvers, MA, USA) Blots were washed and re-incubated with an Actin antibody 1:200 (Sigma-Aldrich, St. Louis, MI, USA), as loading control. Anti-rabbit IgG-HRP 1:2000 (Santa Cruz Biotechnology, Dallas, TX, USA) was used as secondary antibody. Blots were incubated with the Western Blotting Luminol Reagent (Santa Cruz Biotechnology Dallas, TX, USA). Protein levels were analyzed with ImageJ.

### 2.8. ROS Assay

AX4 and mutant cells were incubated in PBS containing 5 µM dihydroethidium (ThermoFisher Scientific, Waltham, MA, USA) for 20 min at 37 °C. Cells were then washed with PBS and analyzed by flow cytometry.

## 3. Results

### 3.1. Generation of D. Discoideum dkc1 Mutant Strains

**Figure 1:**
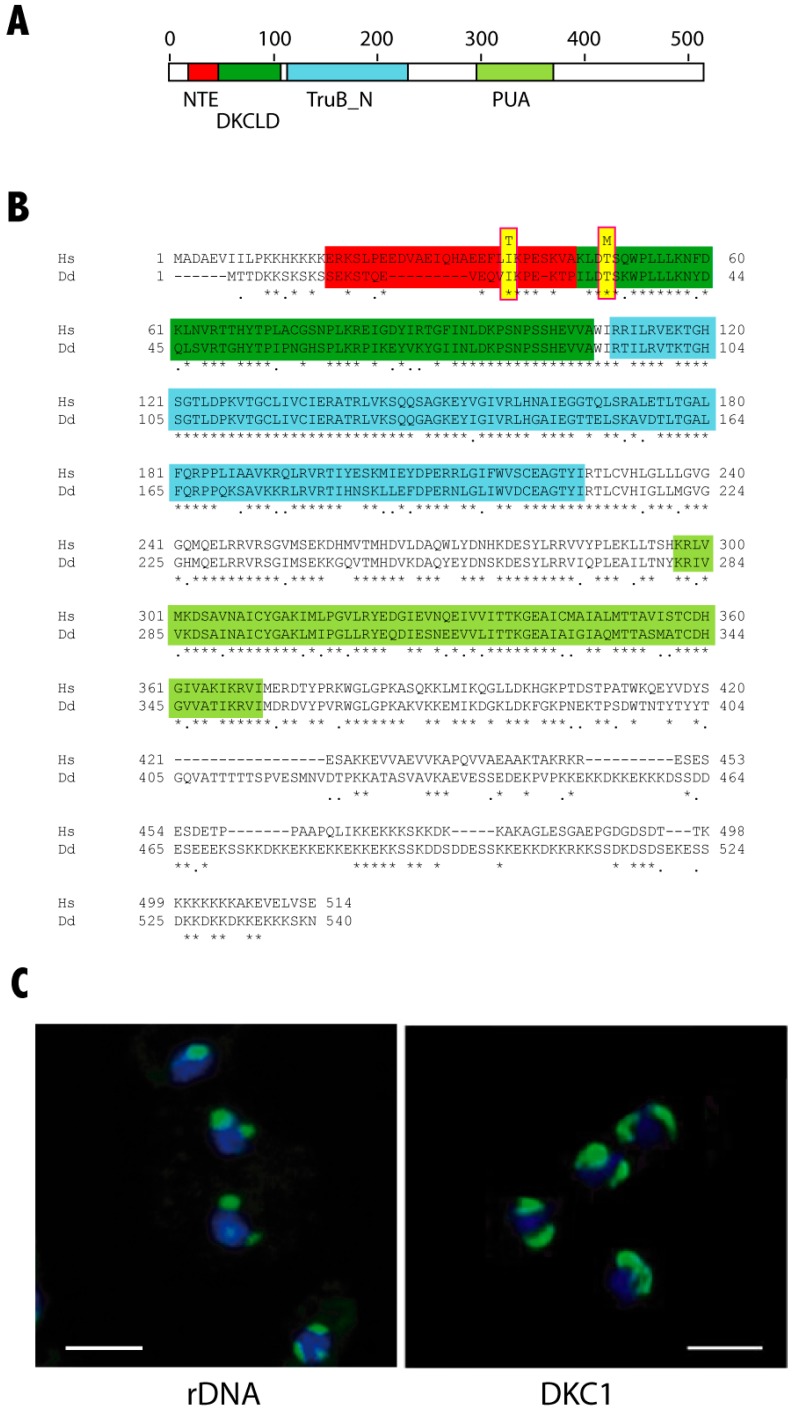
Protein sequence and expression of *D. discoideum* dyskerin, (**A**) Schematic representation of dyskerin conserved domains. The conserved domains N-terminal extension (NTE), dyskerin-like domain (DSKLD), pseudouridin synthase catalytic domain (TruB_N) and RNA-binding domain (PUA) are represented according to the human protein. Amino acid positions are shown in the upper part of the diagram. (**B**) Comparison of the human (Hs) and *D. discoideum* (Dd) dyskerin amino acid sequences. Functional conserved domains are indicated using the same color code as in Panel A. The amino acids mutated in the *D. discodeum* protein in this study are highlighted in yellow. (**C**) *D. discoideum* nucleoli, one or two in each cell, were identified in the left panel by hybridization with a probe specific for the 26S ribosomal RNA. The right panel shows the expression of a *D. discoideum* dyskerin-GFP fusion protein after transfection of the cells with a dyskerin expression vector. Nuclei were stained with DAPI and are shown in blue color. Scale bars correspond to a distance of 5 μm.

The *D. discoideum nola4* gene (nucleolar protein family A member 4) codes for a protein highly similar to human DKC1 (Figure 1A,B). Both proteins present a 78% of similarity and 68% of identity. The functional domains identified in human dyskerin are schematically shown in Figure 1A, including the N-terminal extension (NTE), dyskerin-like domain (DKCLD), the catalytic TruB_N and the RNA-binding PUA domains. The aminoacid sequences of human and *D. discoideum* proteins are compared in Figure 1B where functional domains have been shadowed in the same colors as in Figure 1A. Sequence identity is very high in these functional domains, with the exception of the NTE domain, that shows a deletion in the *D. discoideum* protein. Besides, the *D. discoideum* protein fused to the Green Fluorescence Protein (GFP) localizes to nuclear bodies similar to nucleoli (Figure 1C). These data strongly indicate that the protein coded by the gene *nola4* is the *D. discoideum* dyskerin homologous protein and will be named DKC1 in this article.

**Figure 2:**
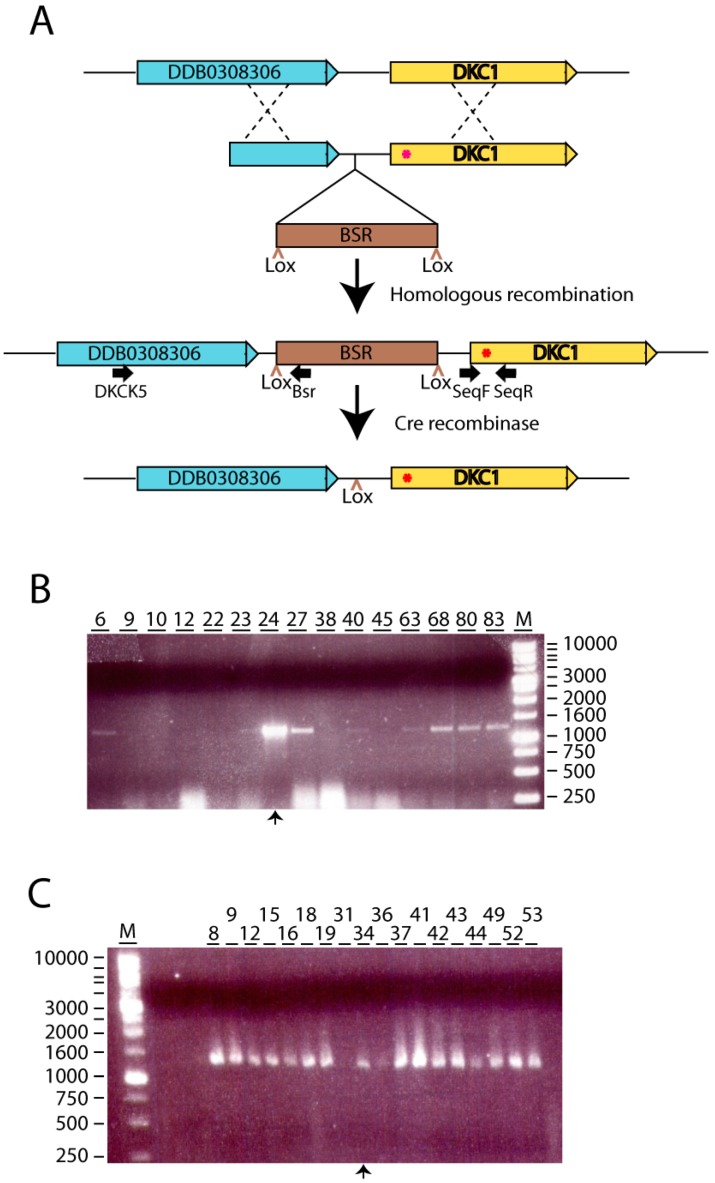
Generation of *dck1* knockin strains, (**A**) Schematic representation of the strategy used for the generation of *dkc1* knockin strains by homologous recombination. The genomic organization of the *dkc1* locus is represented to scale in the upper part indicating the *dkc1* gene, the upstream DDB0308306 gene and the intergenic region that contains the putative *dkc1* promoter region. The vector used for homologous recombination is represented underneath and includes the blasticidin resistance cassette (BSR) flanked by two Lox sites. Mutated residues are represented as red asterisks on the *dkc1* gene. The product of homologous recombination is represented bellow. The location of the oligonucleotides used for verification of homologous recombination is indicated. The Bsr oligonucleotide hybridizes into the blasticidin-resistance cassette while the DKCK5 oligonucleotide correspond to a region of the BBB0308306 genes located upstream of the 5′ arm of the recombination vector. The position of the oligonucleotides used for amplification and sequencing of the mutated region, SeqF and SeqR, is also indicated. The lowest scheme represents the structure of the locus after removal of the BSR cassette upon expression of the Cre recombinase. Only one Lox site and one short region of the cloning vector remain in the intergenic region. (**B**) DNA was isolated from several clones obtained after homologous recombination using the knockin-1 vector and before BSR cassette removal. The insertion of the BSR cassette in the *dkc1* locus was tested by PCR using the oligonucleotides DKCK5 and Bsr, whose location is indicated in Panel A. PCR products were analyzed by agarose gel electrophoresis. The number of each clone is shown in the upper part of the picture. The arrow drown in the lower part of the picture indicates the clone selected for further analysis. The migration of the more relevant fragments of the 1 Kb ladder from Nippon Genetics Europe GMBH (Germany) is shown to the right. (**C**) DNA from clones obtained after homologous recombination using the knockin-2 vector was analyzed for the insertion of the BSR cassette by PCR as indicated in Panel B. Migration of the 1Kb ladder is shown to the left of the panel.

**Figure A1:**
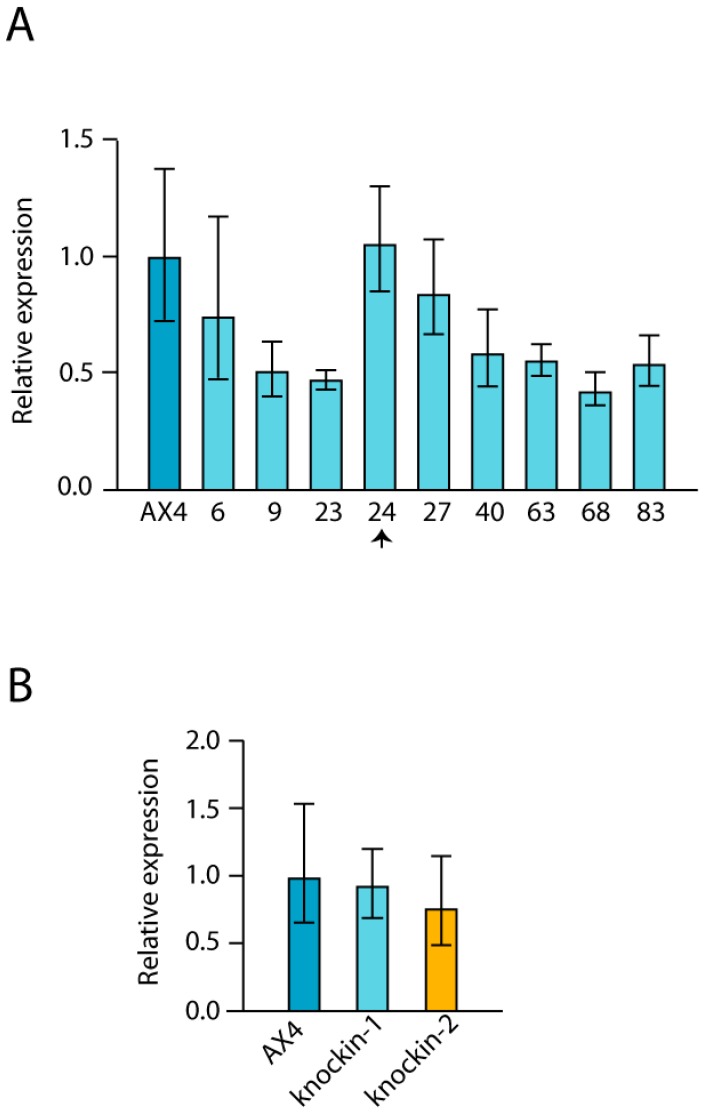
*Dkc1* expression in clones obtained by homologous recombination. RNA was isolated from different clones that had incorporated *dkc1* by homologous recombination, as determined by the PCR test shown in Figure 2. The relative expression of *dkc1* mRNA was determined by reverse transcription and quantitative PCR and compared to that of AX4 cells. Panel **A** shows the results obtained for several clones corresponding to the knockin-1 generation. Clone numbers are shown in the X-axes. The arrow indicates the clone that was selected for further analysis. Panel **B** shows the relative expression of the knockin-1 and knockin-2 clones selected for the study after removal of the Blasticidin resistance cassette upon expression of the Cre recombinase. This result is in agreement with the data on *dkc1* expression shown in Figure 6.

The NTE and DKCLD domains represent one of the two hotspots for X-DC causative mutations [15]. Among the residues of this region identical in human and *D discoideum* DKC1 proteins are two amino acids mutated in DC patients: Isoleucin 38 (Ile38) and Threonin 49 (Thr49) in humans that correspond to Ile23 and Thr33, respectively, in *D. discoideum*. The variants found in these residues (Ile38Thr and Thr49Met) have been described in patients of the more severe DC disease variant, the Hoyeraal Hreidarsson syndrome [34,35]. *D. discoideum* strains containing these two mutations were generated by homologous recombination. In the first step of this process, the *D. discoideum dkc1* gene was amplified by PCR and cloned in the pDV-cGFP-cTAP vector. The expression of the fusion protein was shown in Figure 1C. The mutations Ile38Thr and Thr49Met (according to the human DKC1 amino acid sequence) were independently generated by in vitro mutagenesis. In the second step, mutated genes were cloned as 3′ arm in the vector pLPBLP used for homologous recombination, as detailed in the Materials and Methods section and schematically shown in Figure 2A. The 3′ arm of the construct contained the putative *dkc1* promoter (fragment −358 to +1) and the complete *dkc1* coding region including the termination codon but not the 3′ untranslated region. *D. discoideum* AX4 cells were transfected with these vectors and knockin strains identified by PCR reactions, as shown in Figure 2B,C and by sequencing of the *dkc1* gene. In addition, *dkc1* mRNA expression levels were determined for several recombinant clones, as shown in Figure A1, panel A for the clones corresponding to mutant 1. Two clones showing expression levels similar to that of the wild type AX4 strains were selected (clones 24 and 27 in Figure A1, panel A). In the case of mutation 2, only one clone incorporated the mutation. In a last step, the blasticidin-resistance cassette present in the vector was deleted by expression of the cre recombinase to maintain the genetic structure of the mutated gene. After this screening procedure, clones 24 carrying mutation 1 and clones 34 carrying mutation 2 were selected (knockin-1 and -2 in the rest of the manuscript). The expression levels of *dkc1* in these final clones was similar to that of AX4 cells indicating that *dkc1* expression was not altered as a consequence of the mutations introduced (Figure A1, panel B).

### 3.2. Proliferation and Telomere Structure of dkc1 Mutant Strains

**Figure 3:**
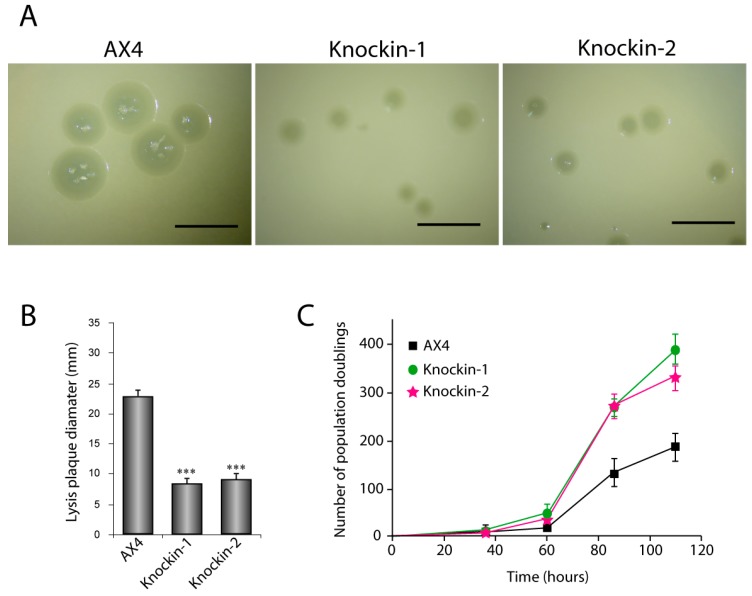
Proliferation capacity of dyskerin-mutated *D. discoideum* strains, D. discoideum AX4 cells were transfected with plasmids containing mutated dyskerin and cells that incorporated the mutations by homologous recombination selected. Knockin-1 strain incorporated the Ile38Thr and the knockin-2 strain de Thr49Met mutation. Wild-type and mutant strains were grown on bacteria for 5 days and the lyses plaques analyzed. (**A**) Panel A shows a picture of the plaques. Scale bar correspond to 5 mm. (**B**) Panel B shows a determination of the mean plaque diameter and the standard deviation determined after measuring 75 plaques from each strain. Statistical significance: *** *p* < 0.001. (**C**) Cell proliferation in liquid culture. 3 × 10^5^ cells from each strain were cultured on axenic, liquid media. At the indicated times the number of cells was determined and the number of population doublings calculated. Mean values and standard deviations of three independent experiments are represented.

*D. discoideum* amoebae can grow in liquid media or on bacterial layers originating clear plaques. The proliferation capacity of *dkc1* mutants was determined in both conditions. The proliferation capacity feeding on bacteria was determined by comparison of the size of the plaques originated by these strains versus those of wild type AX4 cells (Figure 3A). The area of 75 plaques was calculated for each strain (Figure 3B) and showed a significant decrease in both *dkc1*-mutant strains. In liquid culture, the number of population doublings was determined for AX4 and knockin strains during 120 h of culture (Figure 3C). Under these conditions, both knockin strains proliferated to higher rate than AX4 cells. However, no significant alterations were observed during multicellular development of the knockin strains and fruiting bodies similar to those of AX4 were obtained (data not shown).

**Figure 4:**
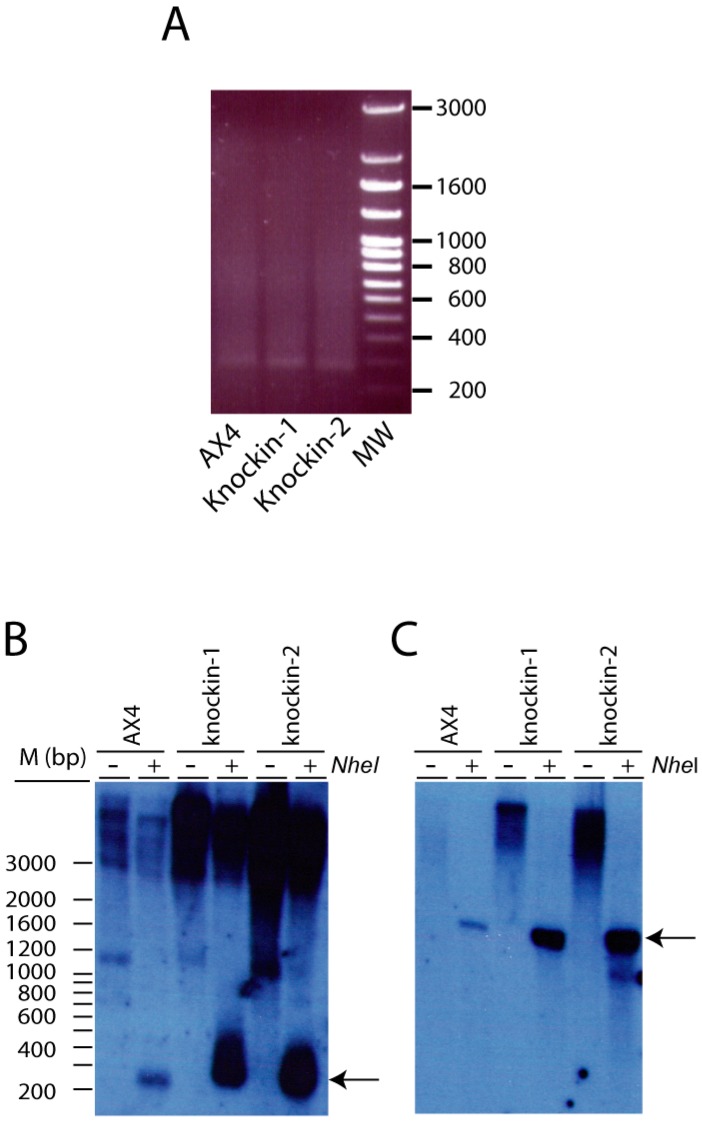
Telomere structure of dyskerin-mutated *D. discoideum* strains, (**A**) Telomere length was determined by PCR reaction using an oligonucleotide probe complementary to the A(G)n repeats of *D. discoideum* telomeres and another complementary to the close subtelomeric region. The migration of the amplification products obtained from DNA isolated from the wild-type (AX4) and mutant strains (Knockin-1 and -2) is shown for one experiment representative of the four made with similar results. The MW line shows the migration of the Ladder VII molecular weight marker (Nzytech, Lisbon, Portugal). The size of some of the markers is shown to the right. (**B**) Telomere structure was analyzed by Southern blot of DNAs obtained from AX4, knockin-1 or knockin-2 strains and either non-digested (−*NheI*) or digested with the *NheI* restriction enzyme (+*NheI*). The panel shows the results obtained after hybridization with a telomere-specific probe. (**C**) The blot shown in panel B was washed and hybridized with a probe complementary to the 26S rRNA. Arrows indicate specific hybridization bands obtained after *NheI* digestion. The migration of Ladder VII molecular weight marker (Nzytech, Lisbon, Portugal) is shown to the left of panels B and C.

DKC plays a fundamental role in telomere elongation as part of the telomerase complex. Therefore, telomere length was determined by PCR reactions in the wild type and *dkc1* mutant strains using an oligonucleotide from the subtelomeric region and another complementary to the A(G)_n_ telomere sequence, as previously described [26]. The results obtained indicated that telomeres from the three strains presented similar length (Figure 4A). Telomere structure was also analyzed by Southern blots after digestion of the DNA with the *Nhe*I restriction enzyme that cuts the DNA at about 250 bp of the telomere end in wild-type cells [26]. An oligonucleotide complementary to the A(G)_n_ telomeric sequenced was used as probe. Similar restriction fragments were obtained in the wild-type and *dkc1*-mutant strains (Figure 4B). Hybridization to a 26SrRNA oligonucleotide was used as control (Figure 4C). The results of these experiments indicate that the tested *dkc1* mutations did not impairs telomere elongation and structure in *D. discoideum*.

### 3.3. RNA Pseudouridylation in dkc1 Mutant Strains

**Figure 5:**
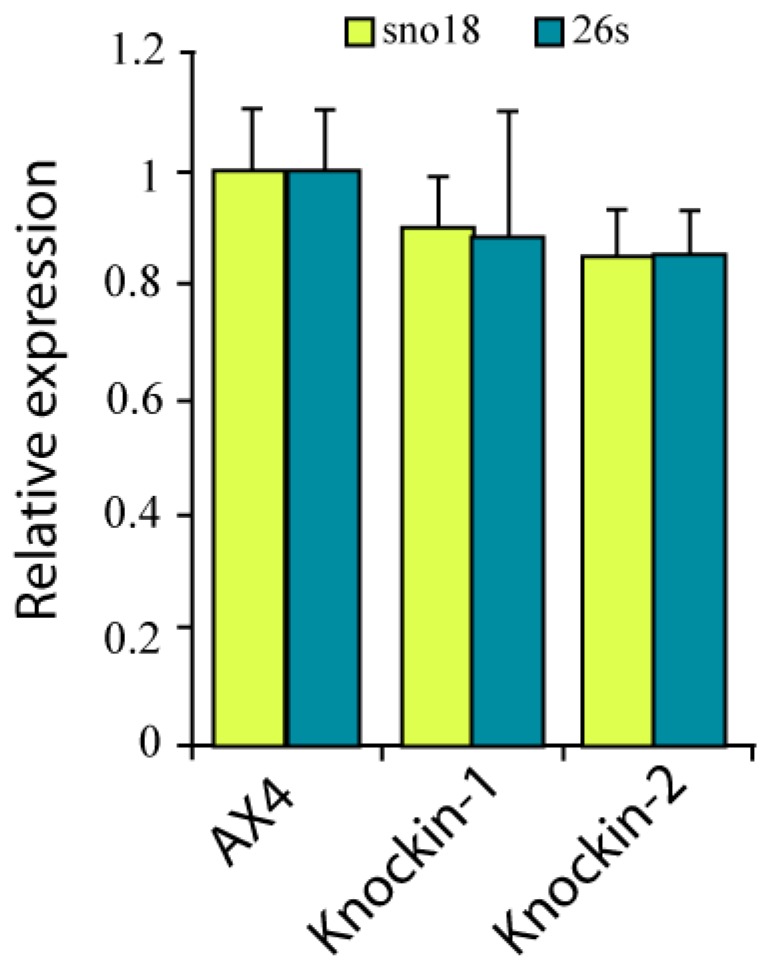
Pseudouridylation activity of *D. discoideum* dyskerin mutants. RNA was purified from wild-type (AX4) or dyskerin mutant (knockin-1, knockin-2) strains and treated, or not, with *N*-cyclohexil-*N*′-(2-morpholinoethyl) carbodiimide metho-*p*-toluenesulphonate (CMCT). The amount of unmodified sno18 (yellow bars) or 26S rRNA (blue bars) was determined by RT-qPCR. The relative amount of RNA determined for treated versus untreated samples was referred to that obtained for AX4 RNA. Mean values and standard deviation obtained in triplicate experiments are represented.

Dyskerin has pseudouridine synthase activity and is required for pseudouridylation of several RNA molecules, including rRNAs and snoRNAs, as mentioned in the introduction section. The possible consequences of *dkc1* mutations on pseudouridylation was studied by treatment of the RNA from these strains with CMCT (*N*-cyclohexyl-*N*′-(2-morpholinoethyl)carbodiimide metho-*p*-toluenesulfonate) that forms adducts between pseudouridines which interfere with reverse transcription of the RNA and can be analyzed by RT-qPCR. Pseudouridylation levels of 26S rRNA and sno18 were studied. Sno18 is the only snoRNA containing H/ACA box identified in *D. discoideum*. The results obtained for the wild-type and the two *dkc1*-mutant strains are shown in Figure 5. No significant differences in pseudoridylation levels were observed indicating that the two mutations analyzed do not affect dyskerin pseudouridin synthase activity.

### 3.4. Expression Levels of GAR1, NHP2 and NOP10 in dkc1 Mutant Strains

**Figure 6:**
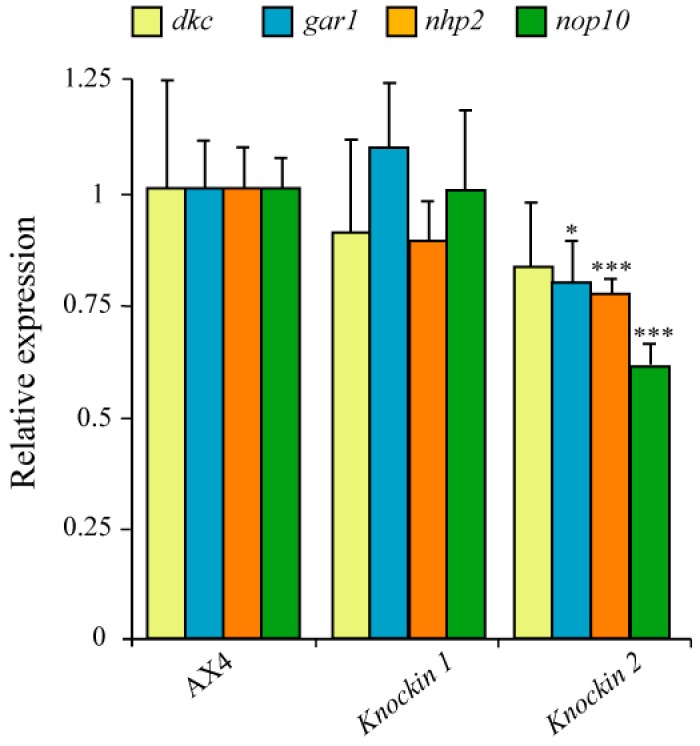
Expression of mRNAs coding for snoRNP component in *D. discoideum* dyskerin mutants. RNA was isolated from Wild-type (AX4) and dyskerin mutant (knockin 1, knockin 2) strains. The expression of the mRNAs coding for the snoRNP components dyskerin (*dkc*), GAR1 (*gar1*), NHP2 (*nhp2*) and NOP10 (*nop10*) was determined by RT-qPCR. Expression data were normalized using the large mitochondrial rRNA as control. Expression values were related to those of the AX4 strain for each gene. Mean values and standard deviations from three independent experiments are represented. * *p* < 0.05; *** *p* < 0.001.

Association of dyskerin to snoRNAs requires the formation of a protein complex composed by GAR1, NHP2 and NOP10, in addition to dyskerin. *D. discoideum* homologous proteins have been identified and the possible consequences of dyskerin mutation on the expression of the coding genes has been studied by RT-qPCR. The results obtained show a modest but significant decrease in *gar1*, *nhp2* and *nop10* mRNA expression in dyskerin mutant 2 but not in mutant 1. Dkc1 expression levels were not significantly different in any of the knockin strains as compared to AX4 (Figure 6).

### 3.5. DNA-Damage Response and Oxidative Stress of dkc1 Mutant Strains

**Figure 7:**
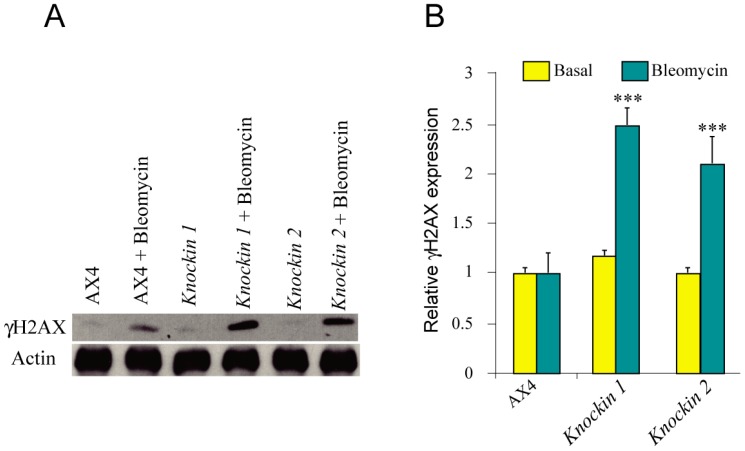
DNA damage response in *D. discoideum* dyskerin mutants. Wild-type (AX4) and dyskerin-mutant (knockin 1, knockin 2) strains were incubated with bleomycin (+bleomycin) for 3 h, or not, and protein extracts were prepared. (**A**) Panel A shows in the upper blot a representative Western blot obtained after incubation with an antibody specific for phosphorylated histone H2AX (γH2AX). The blot was washed and incubated with an anti-Actin antibody as a loading control (lower blot). (**B**) Panel B shows the quantification of the blots from three independent experiments. Samples non-treated with bleomycin are represented in yellow bars, while those obtained from cells treated with bleomycin are represented by blue bars. Mean values and standard deviations are represented. The relative expression obtained for the knockin strains treated with bleomycin was compared to that of treated AX4 cells. *** *p* < 0.001.

Cells of DC patients have been described to activate DNA-response pathways in basal conditions, especially in the presence of DNA-damaging agents such as bleomycin [36]. One of the first steps in DNA-damage response pathways is the phosphorylation of histone H2AX [37]. Therefore, the presence of phosphorylated H2AX (γH2AX) was determined by Western blot without treatment or after incubation of the cells with bleomycin. The results obtained (Figure 7) show a significant increase in H2AX phosphorylation in bleomycin-treated *dck1*-mutant strains.

**Figure 8:**
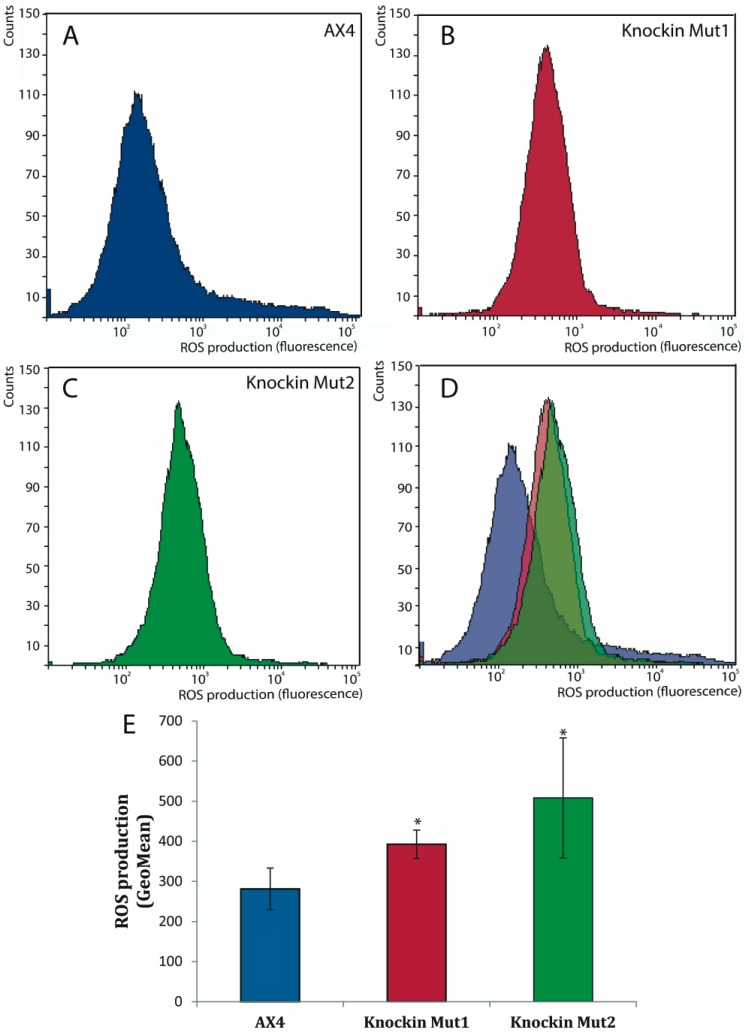
Production of reactive oxygen species by *D. discoideum* dyskerin mutants, (**A**–**D**) Wild-type (AX4) and dyskerin mutant strains (knockin Mut1, knockin Mut2) were collected and 10^6^ cells analyzed for the expression of reactive oxygen species (ROS) by flow-cytometry using the DHE reagent. Representative diagrams obtained for AX4, knockin 1 and knockin 2 cells are represented in panels **A**, **B** and **C**, respectively and overlapped in panel **D**. (**E**) The quantification of the data obtained in three independent experiments is represented. Mean values and standard deviations are shown. * *p* < 0.05.

Another distinct characteristic of cells from DC patients is the increased oxidative stress that can be related to several pathological manifestations such as telomere shortening and cell senescence [38,39]. In order to determine if *dkc1*-mutant strains presented altered levels of oxidative stress, the level of reactive oxygen species (ROS) was determined. Figure 8 shows that both mutant strains presented significantly higher ROS levels than the wild-type strain. These data are in agreement with the increased oxidative stress described in DC patients’ cells and could be related to the increased DNA damage that is also common to patient cells and *D. discoideum dkc1* mutant strains [40].

## 4. Discussion

*D. discoideum* dyskerin mutants have been generated in two evolutionarily conserved residues that reproduce pathogenic variants described in dyskeratosis congenita and Hoyeraal-Hreidarsson patients. This approach was chosen because dyskerin knockout mutants were not obtained in *D. discoideum* after several attempts. Genes coding for DKC1 homologous proteins are essential also in other organisms, as mentioned in the introduction. In male patients, these variants are in hemicygosis since the DKC1 gene is located in the X chromosome. Similarly, the variants are also in hemicygosis in *D. discoideum* cells that grow as haploid organisms. The mutated amino acids (Ile38, Thr49) are in the N-terminal region of the protein that is one of the two domains where more mutations have been found in DC patients [41]. These two residues are very close to residues Lys 39, Lys 43 that are required for dyskerin binding to the telomere RNA (TR), but not to other H/ACA RNAs [15]. Furthermore, residue Ile38 is part of the described DKC1 SUMOylation consensus site [34], while Thr49 is part of the DKC1 binding surface for the H/ACA RNP assembly factor SHQ1 [42]. The T49M mutation enhanced DKC1/SHQ1 interaction and is proposed to reduce the availability of DKC1 for RNP assembly [42]. The other DKC1 region where pathological mutations are more frequent is the C-terminal region. We tried to generate a knockin mutant for A353V, the most frequent DKC1 mutation in DC patients [43], but none of the over 100 recombinant strains analyzed had incorporated the mutation. This result indicates that either this mutation is lethal in *D. discoideum* or that a different recombination strategy is required for the generation of knockin mutations at the C-terminal region of the protein.

The phenotype of the knocking strains for the two mutations analyzed was similar indicating that the possible contribution of secondary mutations is unlikely. Telomere DNA of the mutant strains was analyzed after more than 25 generations of proliferation and no significant changes were observed. The size of the terminal region of A(G)_1–8_ repetitions was similar between the wild type and mutant strains, as determined by PCR amplification and Southern blot analyses. Similar analyses of *terthp*-mutant strains had shown extensive reorganization of the telomere region after 25 generations of proliferation that was more pronounced after additional culture of the strains [26]. These data indicate that the two dyskerin mutations generated in *D. discoideum* do not alter telomere length or structure although the presence of alterations after more prolonged proliferation of the cells cannot be discarded. The analysis of dyskerin mutations in some other model systems has also shown that telomere size did not decrease. For example, in a mouse model of dyskeratosis congenita stem cell ageing was observed in the presence of long telomeres [38,44]. Similarly, a DC model in zebrafish reported undetectable changes in telomerase activity [45]. In addition, regulation of telomeres in *D. discoideum* might not be the same as in human tissues.

Dyskerin is also involved in RNA pseudouridylation and processing, as mentioned above. Alterations in these functions could also contribute to the pathology of DC patients [14]. Defects in RNA processing were observed in the zebrafish model of dyskeratosis mentioned above [45] and in *Drosophila melanogaster* [19]. In the present study no alterations in the pseudouridylation of the 26S rRNA or the sno18 snoRNA were observed.

Besides its role in telomere elongation and in RNA pseudouridylation and processing, other functions have been described for dyskerin (reviewed in [14]). For example, dyskerin has been recently shown to play a role in vesicular trafficking [46], energy metabolism [47] and also in the transcriptional regulation of key pluripotency-related genes [48]. *Drosophila melanogaster* presents atypical telomeres that are not extended by the telomerase complex and has been used to study non-telomeric mechanisms of dyskerin activity. Mutations in the Drosophila dyskerin gene cause developmental delay, defective maturation of rRNA, small body size, alterations in the abdominal cuticle, reduced fertility and impaired somatic stem cell homeostasis [49]. Some of these alterations could be related to DC-patient´s symptoms and have been observed in *D. discoideum*. One of the alterations observed in dyskerin mutants is reduced proliferation when grown on bacteria in contrast with the increased proliferation observed in liquid culture. *D. discoideum* use different feeding mechanisms when growing in both conditions. Bacteria are gobbled by phagocytosis, while liquid media is ingested by macropinocytosis. [50] As mentioned above, dyskerin has been involved in vesicular trafficking and energy metabolism that could be differently involved in phagocytosis and macropinocytosis and also in vesicular trafficking [46]. In addition, feeding on bacteria requires processes such as cell mobility and chemotaxis to bacteria that are not required for macropinocytosis [51]. However, further studies would be required to understand this interesting difference. The results obtained when feeding on bacteria are more similar to those reported in some other model systems. Reduced proliferation has been observed in mammalian dyskerin-mutated cell lines [38] and in induced pluripotent stem cells [52]. Dyskerin mutants in *D. melanogaster* showed altered cell-cycle progression and proliferation, stem cell-growth defects and small body size [49,53].

The other two changes observed in *D. discoideum* mutants, increased levels of ROS and DNA damage response after treatment with bleomycin have also been described in other model systems of DC. Increased oxidative stress and ROS levels have been observed in cells derived from DC patients [39,54] and in a mouse model of dyskeratosis congenita [38]. Expression of a dyskerin isoform that lacks the nuclear localization signal also increased ROS levels in *D. melanogaster* [47].

Increased damage response has been described in bleomycin treated cultured DC cells [39]. Mouse embryonic fibroblasts isolated from a DC mouse model also showed accumulation of DNA damage [38]. The activation of the DNA damage response was independent of telomere length in this mouse model [44]. The increase in DNA damage could be related to the previously discussed elevated ROS levels since they induce the formation of 8-oxo-7,8-dihydro-2′-deoxyguanosin (8-oxo-G) in the DNA producing a DNA-damage response. This reaction is increased in the G-rich telomere regions [55,56]. DNA damage activates the p53 pathway in DC cells inducing cell senescence [57].

## 5. Conclusions

In summary, mutations in *D. discoideum* dyskerin gene that mimic human pathogenic mutations reproduce several aspects of the dyskeratosis congenita disease. This article describes the induction of altered proliferation rates, increased ROS levels and DNA damage signalling in the presence of bleomycin. Alterations in telomere length or structure and RNA pseudouridylation were not observed although more extensive culture time might be necessary for the development of these alterations. *D. discoideum* cells are haploid which resembles the genomic situation of X-linked DC. These characteristics make of *D. discoideum* a good genetically amenable model system for the study of pathological mechanisms and possible therapies for dyskeratosis congenita.

## Figures and Tables

**Table 1:** Oligonucleotides.

Gene	Primer	Sequence (5′-3′)
*dkc1*	DKC1F	GGAAGCTTATGACAACTGGTATGTGTGAAA
	DKC1R	GGTCTAGAATTCTTTGATTTCTTTTTTTCTT
	Mut1dkc1F	CAAGTTACTAAACCAGAAAAAACCC
	Mut1dkc1R	CTGGTTTAGTAACTTGTTCAACTTC
	Mut2dkc1F	CAATTTTAGATATGAGTAAATGGCCATTATTA
	Mut2dkc1R	GCCATTTACTCATATCTAAAATTGGGGTTTTT
	Arm1F	GGGGGCCCGGGTGTTGTAGAGATGCAAGATGG
	Arm1R	GGGTCGACGGATTTCTTGATGAAATACTAGGAG
	Arm2F	GGCTGCAGCTCCTAGTATTTCATCAAGAAATCC
	Arm2R	GGAAGCTTTGTTGAAATTTCTTATTTTAAATAA
	DKCK5	CATCACCATTGAAGAGGGT
	BsR	CCAACCAAGTTTTTTTAAACC
	SeqF	GGAAGCTTATGACAACTGGTATGTGTGAAA
	SeqR	GGAATTGGTGTATAATGACCAG
	PCRDKC1F	GATGACTCTGATGATGAATCATCC
	PCRDKC1R	CCCACAAATTTATAAAGTTTATTTTTATTAATTAG
*sno18*	Sno18F	CAGCCTGCCTCCGTTTTGTGTTTG
	Sno18R	GATTACAGATTGATTAAAGGCACATGTATG
*26S rRNA*	26srRNAF	CCGCTGAACTTAAGCATATCAGTAAGC
	26srRNAR	GCAGTCACAACAGCGGGCTCC
*gar1*	Gar1F	CAGTTGATGAAATTTTCGGACC
	Gar1R	GGTACTTTAGCAATTGGTTTTGG
*nhp2*	Nhp2F	GGCGATGTCAGCCCAATCG
	Nhp2R	GTTGATGAAGCAGTACCTAAAG
*nop10*	Nop10F	GTACTATAACGACAAAGATGGCC
	Nop10R	CCAAATCTCTTCTTTAATGCAATTC
*lmtrRNA*	*lmtrRNAF*	GGGTAGTTTGACTGGGGCGG
	*lmtrRNAR*	CACTTTAATGGGTGAACACC
